# Psychometric properties of a Chinese version of the Fraboni scale of ageism: evidence from medical students sample

**DOI:** 10.1186/s12909-020-02111-7

**Published:** 2020-06-17

**Authors:** Jun-Yao Fan, Hui-Min Zhao, Yue-Ting Liu, Ling-Lin Kong, Jing Mao, Jie Li

**Affiliations:** School of Nursing, Tongji Medical College, Huazhong University of Science and Technology, 13 Hangkong Rd., Qiaokou District, Wuhan, 430030 Hubei Province China

**Keywords:** Ageism, Fraboni scale of ageism, Discrimination, Reliability, Validity, Medical students

## Abstract

**Background:**

The increasing of older adults has led to enormous demand for medical care. However, as a group with unique needs and characteristics, older adults are often discriminated against in the medical field. In this paper, we aimed to translate the Fraboni Scale of Ageism (FSA) into Chinese and examine its construct validity, content validity, and reliability in Chinese mainland medical students. In order to evaluate the prevalence of ageism in Chinese medical students and prompt medical college to adopt necessary teaching methods to mitigate ageism in medical students.

**Methods:**

By Brislin’s translation guidelines, FSA was translated to Chinese. The convenient sampling method was used to select samples for this survey, including 1,974 students from two medical schools in central and north China. Construct validity was verified by the exploratory and confirmatory factor analysis. The content validity index (CVI) was used to assess content validity. The Cronbach’s alpha coefficients and intraclass correlation coefficient (ICC) were used to estimate reliability.

**Results:**

The alpha coefficients for FSA (Chinese version) was 0.81 and ICC was 0.87. The CVI was 0.93. Three factors were identified by exploratory factor analysis explaining 34.84% of the total variance and a three-factor model was confirmed to fit by confirmatory factor analysis.

**Conclusions:**

FSA (Chinese version) is a reliable and valid scale for measuring discrimination degree against older adults in Chinese medical students.

## Background

With the development of world economy and medical technology, average life expectancy has been extended and the aging of population has become a global phenomenon rapidly. The World Health Organization reported the number of people over 60 will rise to 2 billion by 2050 [1]. Now more and more countries has entered the aging society all around the world. In Chinese mainland, situation is even more serious. Data from the National Bureau of Statistics [2], the number of older people over 60 in China has reached 250 million, accounting for 17.9% of the total population by the end of 2018.

Ageism has become a ripe research topic for scholars in the context of global population ageing. People are accustomed to associate the older adults with many negative traits [3]. In 1960, Butler first used the concept “ageism” to express prejudice and discrimination toward older adults only because they are old [4]. Then Palmore defined ageism in terms of individual’s cognitive and affective towards older adults [5]. The Encyclopedia of China defines ageism as the prevailing prejudice against older adults and the resulting behaviors [6]. In a word, ageism includes people’s jaundiced attitudes, emotions, and behaviors towards older adults.

According to literature, the identified attitudes of medical students or workers towards older adults varied greatly in different countries [7]. In a Spanish university, 54% final year medical students held a positive attitude towards older adults [8]. Faronbi reported 66.1% nursing students in Nigeria had a positive attitude [9]. The health care professionals also showed generally positive attitude to older patients [10]. However, there were several studies reported negative attitudes among medical students toward older adults [11, 12]. Rathnayake reported nearly half of nursing students in a Sri Lankan nursing school had negative attitude to older adults [11]. In an Australian university, 87.5% nursing students admitted negative behaviors to older adults [12]. Compared to other country, the English article about medical students’ attitude towards older adults in China is insufficient, and the prevalence of ageism among Chinese medical students remains unclear. Moreover, influenced by the traditional culture of Chinese ancestors advocating filial piety, it is generally believed that Chinese people have a low degree of ageism. But contrary to the common view, studies revealed that Chinese young people actually hold more negative attitudes toward older adults compared to American and British youth [13, 14]. Will the same phenomenon occur among Chinese medical students? Therefore, it is essential to acquire an applicable and effective measurement with Chinese context, which could provide feasibility of conducting study to demonstrate status quo regarding ageism among medical students.

There are several tools frequently-used to measure the prevalence of ageism among medical students [15]. Kogan’s Attitudes toward Old People Scale (KAOP) contains 34 items and adopts 6-point Likert, with negative to positive responses [16]. Study confirmed that KAOP had good reliability and validity [17]; Facts on Aging Quiz (FAQ), an indirect measure of negative and positive ageism, includes 25 true-false items, mainly used to measure individuals’ knowledge of some factual knowledge on physiological, psychological and social roles during aging [18]. Therefore, the scale cannot measure the extent of ageism directly. The Fraboni Scale of Ageism (FSA) was prepared based on the concept of ageism proposed by Butler [19]. It is commonly used to evaluate the cognitive status of ageism, containing 29 items. Among above instruments, KAOP mainly focuses on assessment of stereotype of older adults; FAQ evaluates the personal knowledge of older adults reflecting the attitude of older adults; FSA assesses both cognitive and affective aspect of ageism, making up of three multidimensional constructs: antilocution, avoidance and discrimination and getting a comprehensive measurement of ageism intuitively [19, 20]. It is helpful to evaluate the cognition and emotion of medical students towards older adults, which can more comprehensively measure the degree of ageism and reduce the prevalence of ageism in medical filed [15].

In 1990, the Fraboni Scale of Ageism (FSA) was developed by Fraboni in Canada, and its reliability and validity were satisfying among Canadians: Cronbach’s alpha coefficients for FSA was 0.86 suggesting the scale is homogeneous; the items all loaded (> 0.40) on their respective factors suggesting some stability in the three factor structure [19]. In 2005, Rupp et al. further explored factor structure of FSA in the United States. Their results revealed a three-factor structure that was somewhat different from the one suggested by Fraboni et al., and they conducted a confirmatory factor analysis to further validate this revised three-factor model, results showed a significant improvement in fit over the original structure [20]. In 2008, Bodner et al. generalized FSA to Israeli students. Results showed that the internal consistency reliability and structure validity of FSA were acceptable [21]. In 2012, Kutlu et al. assessed the reliability, validity, and psychometric properties of the Turkish version of FSA [22]. Results showed the content validity index for FSA was 0.98, the a coefficient was 0.84, and the split-half reliability was 0.81, the three factors represented 38.31% of the variance. They concluded that the Turkish version of FSA is a suitable instrument for measuring ageism in the Turkish population. However, so far there have not been any research about validity and reliability assessment of FSA in Chinese medical students. Therefore, the purpose of the study was to adapt FSA to Chinese language and culture, then assess its reliability and validity in Chinese medical students.

## Methods

### Participants

The study adopted convenient sampling method, recruiting participants among first- to fifth-year medical students in China between May 1 and May 30, 2019. The study took place at Fenyang College of Shanxi Medical University and Tongji Medical College, Huazhong University of Science and Technology, located in Fenyang Shanxi province and Wuhan Hubei province with approximately 6400 and 4100 undergraduate students, respectively. Inclusion criteria were following: a full-time medical student in school, informed consent, voluntary to participate in this investigation. Exclusion criteria were: students returning to school after working first or providing incomplete responses in the questionnaire. A sample size of 1000 for factor analysis is recommended as ideal [23], we need 1000 for exploratory factor analysis and 1000 for confirmatory factor analysis. So, the initial sample included a total of 2034 students, of which 1974 students completed the whole set of questionnaire, the effective response rate of questionnaire was 97.05%.

### Instrument

#### Demographic information

Demographic information including age, gender, grade and department.

#### The Fraboni scale of ageism

FSA was developed by Fraboni, including 29 items [19]. The items were responded by a likert scale, ranging from 1 (strongly disagree) to 4 (strongly agree). It’s worth noting that item numbers 8, 14, 21, 22, 23, 24 are positive statements and scores should be reversed. Total scores range 29 to 116, the higher the score, the greater the ageism. An exploratory factor analysis found FSA measure three levels of prejudices: antilocution, avoidance, and discrimination [19]. The questionnaire took approximately 5 min to complete.

### Procedures

This study proposed to evaluate validity and reliability of the Chinese version of FSA in medical students. We conducted the study in two steps: translated and validated scale. All translation processes was conducted based on modified Brislin’s translation model [24]. In the forward-translation, we invited two bilingual translators who are Chinese and had studied in the United States ever to translate the FSA to Chinese independently. In back-translation, we invited two professional bilingual translators who is blind to FSA to translate back to English (from Chinese to English). Then they had a meeting to look back to the back-translations, find differences in meaning to achieve the most refined culturally equivalent meaning. Until the members were all agreement on the culturally same meaning in the two version of the FSA, the meeting wasn’t ended. Then we selected 20 medical students for the pre-survey by the FSA (Chinese version) and collected their opinions to make the scale items clear and easy to understand. Before data collection, we invited six experts (2 geriatric nurse clinical specialist, 2 health care providers who work at nursing home, 2 associate professors whose research interest is elderly abuse) to evaluate the content validity of the FSA (Chinese version) based on relevance as 1 (high relevant), 2 (quite relevant), 3 (somewhat relevant), and 4 (not relevant).

### Data collection

The study was approved by the Institutional Review Board of Tongji Medical College, Huazhong University of Science and Technology. Trained investigators recruited eligible subjects at Fenyang College of Shanxi Medical University and Tongji Medical College, Huazhong University of Science and Technology. For students from Tongji Medical College, Huazhong University of Science and Technology, investigators obtained written inform consent and collected information from all the participants who filled in the questionnaires by themselves. The data collection procedure for students from Fenyang College of Shanxi Medical University was as follows: First, one author sent an email to the investigator who is a nursing faculty member in this university. The email described the purpose, content and eligibility criteria of this study, and attached a link to the online questionnaire. Second, the email was sent to the undergraduate medical students by the investigator, then the students finished the survey online. In order to prevent repetition, we designed it so the same mobile phone number could only fill in the questionnaire once and the questionnaire could only be submitted when it was completely filled out. In addition, it is recommended the minimum sample size for test-retest reliability is 15 subjects [25]. So, 30 subjects were selected to fill in the same questionnaires 4 weeks following the first time for filling out the questionnaire for assessment of the test–retest reliability. Before the survey, students were informed that participation was voluntary, anonymous and confidential. If medical students agreed to participate this study, they signed a consent form and then completed the questionnaires.

### Statistical analysis

The Statistical Package for Social Sciences, version 21.0 (SPSS IBM Corp), was performed statistical analyses except confirmatory factor analysis (CFA). The CFA was carried out using SPSS Amos, version 21.0. Content validity was evaluated by the content validity index (CVI). The reliability was assessed by Cronbach’s a coefficients and acceptable level was set at 0.70 [26]. As for test-retest reliability, it was determined by computing the intraclass correlation coefficient (ICC), the minimal acceptable value was set at 0.60 [27]. The exploratory factor analysis (EFA) and CFA were used to perform construct validity. First, the data is divided into two parts, 967 samples for EFA and 1007 samples for CFA. When the value of Kaiser-Meyer-Olkin (KMO) is > 0.60 and Bartlett’s test of sphericity is significant, the samples are appropriate for factor analysis. The number of factors was determined by eigenvalues > 1 and scree plot. Factor loading > 0.30 were considered appropriate. The model fitness was performed by CFA. Factor loading reach a significant level the chi square degree of freedom ratio (CMIN/DF) < 3, the goodness-of-fit index (GFI) > 0.90, the incremental fit index (IFI) > 0.90, the compare fitting indices (CFI) > 0.90, and the root-mean-square error of approximation (RMSEA) < 0.08 indicated the model fit the hypothesized model well [28].

## Results

### Demographic data

A total of 1974 medical students completed the questionnaire in this study, of whom 23.5% (463) were freshman, 35.1% (693) were sophomore, 28.7% (566) were junior, 8.9% (176) were senior, the rest 3.9% (76) were the fifth year students. The mean respondents’ age was 19.91 ± 1.52 years. Of the 1974 respondents, 66.7% (1317) were female; 28.5% (562) were from School of Clinical Medicine, 29.5% (583) were from School of Basic Medicine, 7.3% (145) were from School of Pharmacy majoring in pharmacy, 20.2% (399) were form School of Nursing majoring in nursing, 7.3% (144) were form School of Public Health majoring in preventive medicine, 3.9% (77) were form School of Medicine and Health Management, while 3.2% (64) were from Department of Forensic majoring in forensic.

### Validity

#### Content validity

The CVI was used to assess items validity [29]. Six experts were asked to rate each item of the FSA (Chinese version) based on relevance as 1 (high relevant), 2 (quite relevant), 3 (somewhat relevant), and 4 (not relevant). A CVI was computed as the number of items giving a rating of either 1 or 2, divided by the total number of items. The CVI ranged 0.83 from 1.00 and the total CVI was 0.93, indicating content validity was acceptable.

#### Construct validity

**Fig. 1:**
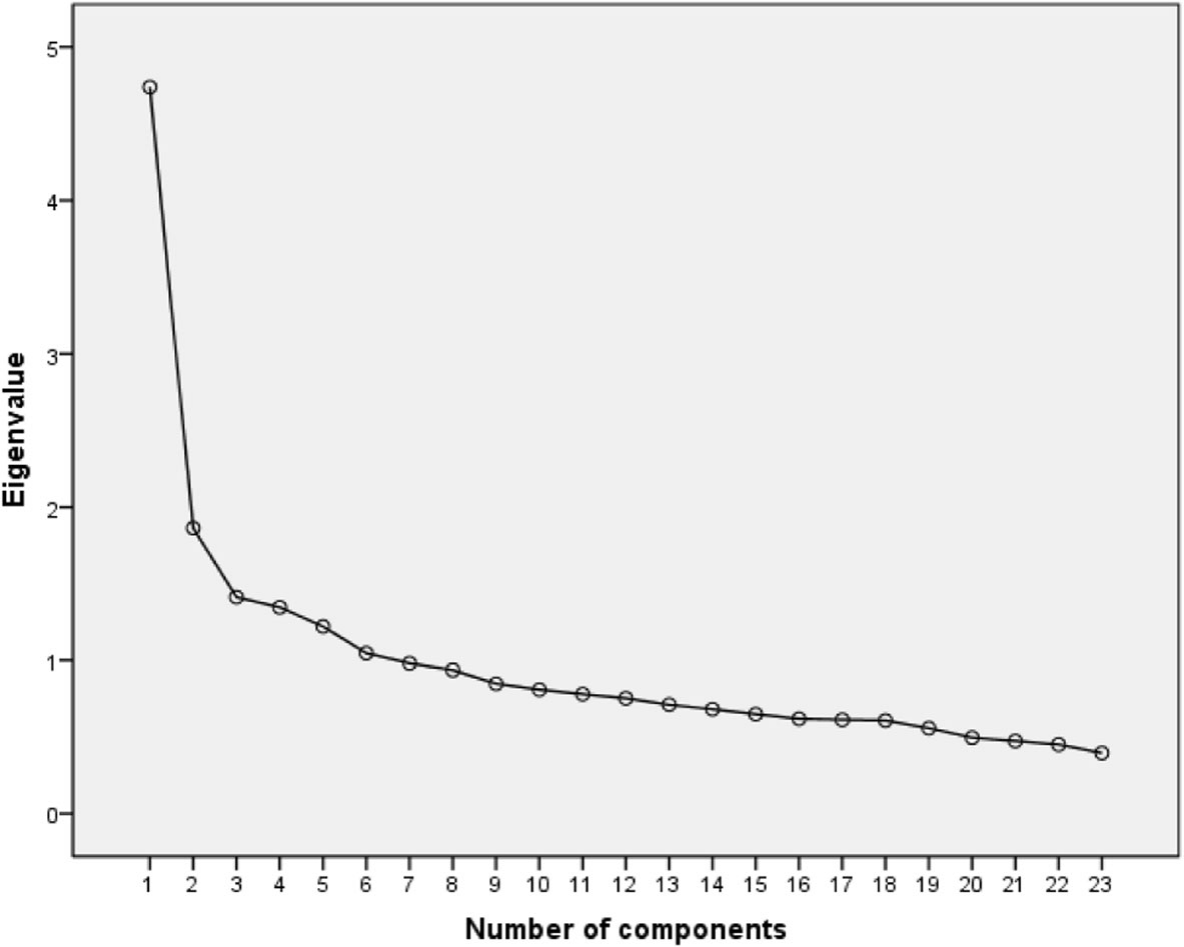
Scree plot of the Chinese version of Fraboni Scale of Ageism

**Fig. 2:**
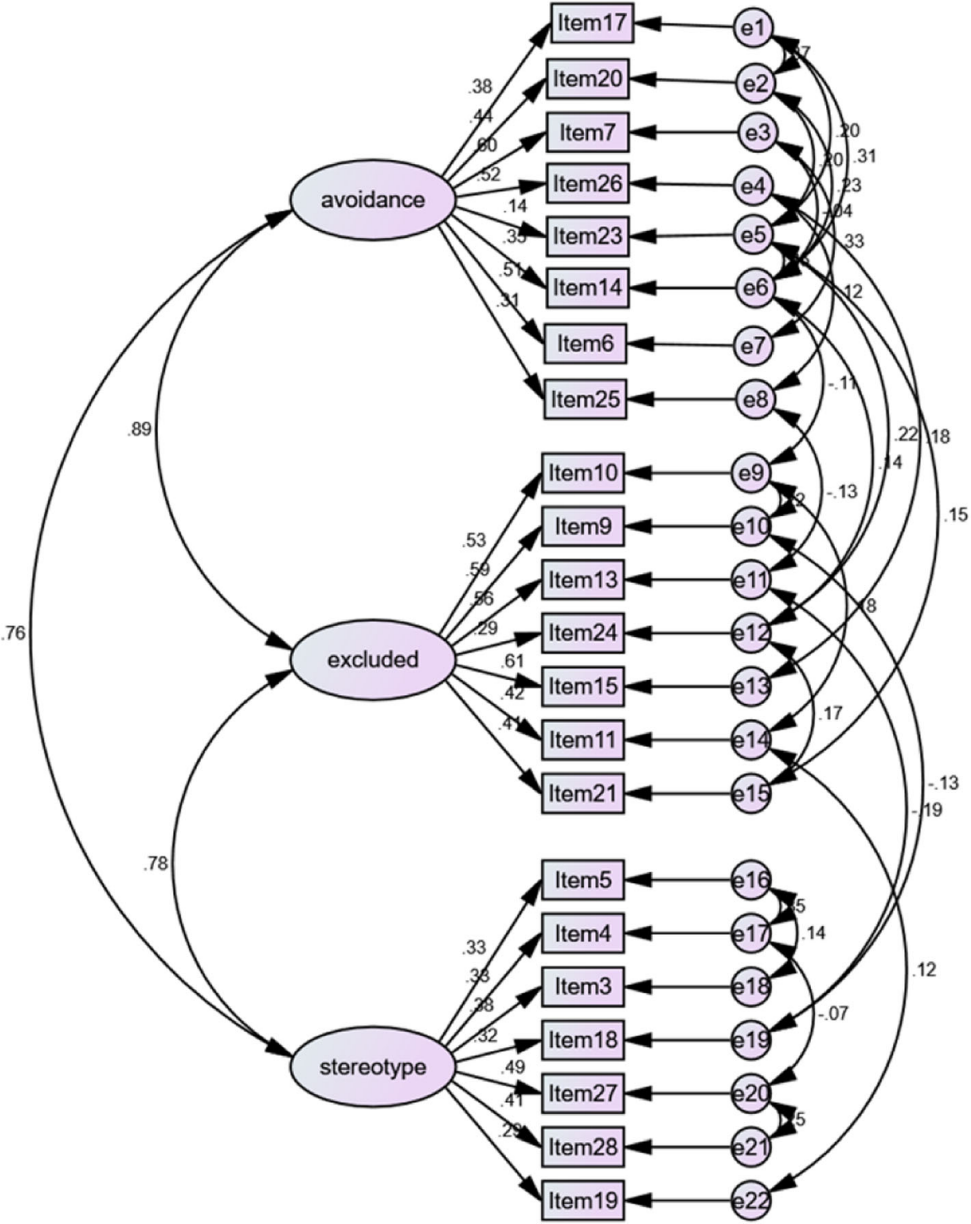
A confirmatory factor model of the Chinese version of Fraboni Scale of Ageism

Principal component analysis and Varimax rotation method (for 23 items) were used in the EFA. The KMO = 0.864 and Bartlett’s test of sphericity (*P* < 0.001) reached statistical significance, supporting factor ability of the correlation matrix. The first factor analysis, we found six factors with eigenvalues above 1, accounting for 50.55% of the cumulative variance. But the curve flattened after the third factor in scree plot (Fig. 1). Therefore we decided to choose three factors, same as the original FSA. Factor analysis was repeated and the number of factors was limited to 3. Three factors explained 34.84% of the variance. The items contained in the three factors were somewhat different from the original scale. Factor 1 consisted of eight items, that showed reluctant to interact with older people, so labeled “avoidance”. Factor 2 consisted of seven items, that showed rejective to accept older people in activities, labeled “excluded”. Factor 3 consisted of seven items, that showed bad impression of old people, labeled “stereotype”. The results of the exploratory factor analysis are showed in Table 1. Item 16 was removed because the load on each factor was < 0.3. Then the three-factor model was tested by CFA. The results of CFA were shown in Fig. 2. The initial model was modified according to the Modification Indices. In the revised model, the CMIN\DF was 2.595; GFI was 0.959; IFI was 0.929; CFI was 0.928, indicating the model fit the data well. The results of EFA and CFA showed that structural validity of FSA (Chinese version) was good.

**Table 1:** Item loading for principal component factor analysis and original scale dimension (*n* = 967)

Item no.	Factor and loading ^a^			Original scale dimension
	Avoidance	Excluded	Stereotype	
Item 17	**0.684**	0.082	0.095	discrimination
Item 20	**0.676**	0.048	0.061	discrimination
Item 7	**0.526**	0.302	0.257	avoidance
Item 26	**0.480**	0.162	0.257	avoidance
Item 23	**0.468**	0.204	−0.220	discrimination
Item 14	**0.468**	0.204	−0.224	avoidance
Item 6	**0.418**	0.337	0.337	avoidance
Item 25	**0.388**	0.001	0.383	anticlocation
Item 10	0.071	**0.704**	0.247	avoidance
Item 9	0.054	**0.693**	0.253	anticlocation
Item 13	0.147	**0.620**	0.044	avoidance
Item 24	0.183	**0.491**	−0.170	discrimination
Item 15	0.362	**0.478**	0.245	avoidance
Item 11	−0.020	**0.446**	0.256	avoidance
Item 21	0.282	**0.405**	−0.104	discrimination
Item 5	−0.136	0.159	**0.617**	anticlocation
Item 4	−0.151	0.076	**0.600**	anticlocation
Item 3	0.137	0.008	**0.563**	anticlocation
Item 18	0.374	−.006	**0.444**	discrimination
Item 27	0.195	0.289	**0.373**	anticlocation
Item 28	0.287	0.153	**0.364**	anticlocation
Item 19	0.000	0.092	**0.358**	anticlocation
Item 16	0.175	0.018	0.284	

Extraction method: Principal component analysis

Rotation method: Kaister standardized orthogonal ratation

^a^Rotation converges after ten iterations

### Reliability

As it is showed in Table 2, six items (1, 2, 8, 12, 22, 29) of the FSA had low total correlation of items and also decline total α coefficient of the scale. These items were recommended to be removed. In addition, item 16 was excluded in factor analysis. Therefore, the final Chinese version scale include 22 items. The internal consistency reliability α coefficient for these 22 items was 0.81 and test-retest reliability ICC was 0.87, indicating that internal consistency reliability and retest reliability of FSA (Chinese version) were acceptable.

**Table 2:** Corrected item-total correlation and Cronbach’s Alpha statistics of Fraboni Scale of Ageism (*n* = 1974)

Items	Corrected item-total correlation	Cronbach’s Alpha if item deleted
1. Teenage suicide is more tragic than suicide among the elderly	0.143	0.755
2. There should be special clubs set aside within sports facilities so that the elderly can compete at their own level	−0.238	0.774
3. Many elderly people are stingy and hoard their money and possessions	0.305	0.745
4. Many elderly people are not interested in making new friends, preferring instead the circle of friends they have had for years	0.255	0.748
5. Many elderly people just live in the past	0.302	0.745
6. I sometimes avoid eye contact with elderly people when I see them	0.454	0.737
7. I don’t like it when elderly people try to make conversation with me	0.479	0.736
8. Elderly people deserve the same rights and freedoms as other members of our society	0.104	0.758
9. Complex and interesting conversation cannot be expected from most elderly people	0.481	0.734
10. Feeling depressed when around elderly people is probably a common feeling	0.457	0.735
11. Elderly people should find friends their own age	0.361	0.742
12. Elderly people should feel welcome at social gatherings of young people	−0.138	0.770
13. I would prefer not to go to an open house at a seniors club if invited	0.409	0.739
14. Elderly people can be very creative	0.262	0.747
15. I personally would not want to spend much time with an elderly person	0.509	0.732
16. Most elderly people should not be allowed to renew their drivers licenses	0.232	0.749
17. Elderly people don’t really need to use our community sports facilities	0.345	0.743
18. Most elderly people should not be trusted to take care of infants	0.302	0.745
19. Many elderly people are happiest when they are with people their own age	0.215	0.750
20. It best that elderly people live where they won’t bother anyone	0.360	0.742
21. The company of most elderly people is quite enjoyable	0.296	0.746
22. It is sad to hear about the plight of the elderly in our society these days	0.073	0.757
23. Elderly people should be encouraged to speak out politically	0.207	0.750
24. Most elderly people are interesting, individualistic people	0.259	0.747
25. Most elderly people would be considered to have poor personal hygiene	0.309	0.744
26. I would prefer not to live with an elderly person	0.407	0.739
27. Most elderly people can be irritating because they tell the same stories over and over again	0.391	0.740
28. Elderly people complain more than other people	0.346	0.743
29. Elderly people do not need much money to meet their needs	0.077	0.759

## Discussion

In the coming decades, China will face unparalleled challenges prompted by aging of population. Medical care for older adults is in great demand. However, ageism is a widespread phenomenon in Chinese society, especially in the medical field [30]. As a form of exclusion in the social life, ageism negatively affects the physical and mental health of older adults. A meta-analysis reviewing data from 32 articles drew a conclusion that being stereotyped negatively affected older adults’ cognitive performance and memory [3]. Different from ageism in the general population, ageism of medical students might change their attitudes to older patients, against establishing a good relationship between medical staff and patients [11]; even impact medical treatment for older patients in their future medical care work, reducing the curative of older patients [31], such as differential treatments providing by medical staff in older cancer patients [32]. Most importantly, ageism among medical students’ willed reduce their willingness to engage in gerontology [7]. Reducing ageism of medical students is not only beneficial to the health of the elderly, but also conducive to social development [33]. Therefore, it is very important to measure degree of ageism in medical staff and take early intervention. To improve the situation, we translated FSA from English to Chinese and assessed the reliability and validity among medical students, so that relevant medical institutions and schools can use FSA (Chinese version) questionnaire to investigate the degree of ageism among medical students, and intervene in advance according to measuring results, hoping to reduce ageism in the medical field.

According to result of reliability test, six items (1, 2, 8, 12, 22 and 29) were recommended to be deleted from original FSA scale, because of low total correlation of items. This was similar to the Turkish version of the FSA, which also removed several items that were inconsistent with local policies, culture and family patterns [22]. After removing the above six items, ICC and α coefficient indicated the scale was reliable.

The CVI was 0.93, indicating content agreement among the six experts on of FSA (Chinese version). EFA and CFA were conducted to test the construct validity of FSA (Chinese version). In the EFA, three factors were found: avoidance; excluded and stereotypes, with which contributing 12.26, 11.58, 11.00%, respectively. Although, the eigenvalues of six factors were above 1 in the first EFA, we thought it is difficult to make them meaningful and name them. Therefore, according to the characteristics of the scree plot, we chose to extract three factors, the same as the original scale. But, the factor structure in our study was a little different from original study conducted by Fraboni [19]. For comparison, factor structure of original and present study was included in Table 1. Also, the factor structures of version of American, Israeli and Turkish were different from each other. This is closely related to the language and culture of each country. In the CFA, three-factor model of FSA (Chinese version) was validated well. By referring to relevant literature and combining Chinese language and culture characteristics, we labeled the three factors in Chinese as “避免; 排除和刻板印象”.

As mentioned above, the adaptation studies of FSA have already many versions of the language, but no Chinese version. There were a few disparity between the health care system, social structure, language, and cultures in different countries, leading to some distinctions between the different versions in structure. This paper does not use any unusual or novel methods and performed a usual validation study for FSA (Chinese version) with sound methods. The structure validity of FSA (Chinese version) is great by the cross-validation with both exploratory and confirmatory techniques. Researchers in China could apply this validated FSA (Chinese version) in other population samples. There are also several limitations in this study. First, the use of a convenient sampling method limits the capacity to generalize the study. Second, only construct validity by factor analysis was tested the instrument. The validation of instrument validity requires a variety of ways, such as criterion-related validity. Moreover, the dimensions of FSA (Chinese version) are somewhat different between the original scale. Therefore, future studies of FSA (Chinese version) including convergent/divergent validity and item analysis are required.

## Conclusion

Internal consistency reliability, test-retest reliability, content validity, and construct validity support FSA (Chinese version) as a valid and reliable instrument for measuring ageism of medical students in Chinese mainland. It is suggested FSA (Chinese version) could be applied to medical students in Chinese mainland.

## Data Availability

Data used during the study are available from the corresponding author on reasonable request.

## Abbreviations

FSA: Fraboni Scale of Ageism
CVI: Content validity index
ICC: Intraclass correlation coefficient
KAOP: Kogan’s Attitudes toward Old People Scale
FAQ: Facts on Aging Quiz
CFA: Confirmatory factor analysis
EFA: Exploratory factor analysis
KMO: Kaiser-Meyer-Olkin
CMIN/DF: The chi square degree of freedom ratio
GFI: The goodness-of-fit index
IFI: The incremental fit index
CFI: The compare fitting indices
RMSEA: The root-mean-square error of approximation
